# Influence of the capping of biogenic silver nanoparticles on their toxicity and mechanism of action towards *Sclerotinia sclerotiorum*

**DOI:** 10.1186/s12951-021-00797-5

**Published:** 2021-02-24

**Authors:** Mariana Guilger-Casagrande, Taís Germano-Costa, Natália Bilesky-José, Tatiane Pasquoto-Stigliani, Lucas Carvalho, Leonardo F. Fraceto, Renata de Lima

**Affiliations:** 1 Laboratory for Evaluation of the Bioactivity and Toxicology of Nanomaterials, University of Sorocaba, Sorocaba, São Paulo, Brazil; 2 Laboratory of Environmental Nanotechnology, São Paulo State University, Sorocaba, São Paulo Brazil

**Keywords:** Silver nanoparticles, Biogenic synthesis, Capping, *Trichoderma harzianum*, Hydrolytic enzymes, SDS-PAGE, FTIR

## Abstract

**Background:**

Biogenic nanoparticles possess a capping of biomolecules derived from the organism employed in the synthesis, which contributes to their stability and biological activity. These nanoparticles have been highlighted for the control of phytopathogens, so there is a need to understand their composition, mechanisms of action, and toxicity. This study aimed to investigate the importance of the capping and compare the effects of capped and uncapped biogenic silver nanoparticles synthesized using the filtrate of *Trichoderma harzianum* against the phytopathogenic fungus *Sclerotinia sclerotiorum*. Capping removal, investigation of the composition of the capping and physico-chemical characterization of the capped and uncapped nanoparticles were performed. The effects of the nanoparticles on *S. sclerotiorum* were evaluated in vitro. Cytotoxicity and genotoxicity of the nanoparticles on different cell lines and its effects on nontarget microorganisms were also investigated.

**Results:**

The capped and uncapped nanoparticles showed spherical morphology, with greater diameter of the uncapped ones. Functional groups of biomolecules, protein bands and the hydrolytic enzymes NAGase, β-1,3-glucanase, chitinase and acid protease from *T. harzianum* were detected in the capping. The capped nanoparticles showed great inhibitory potential against *S. sclerotiorum*, while the uncapped nanoparticles were ineffective. There was no difference in cytotoxicity comparing capped and uncapped nanoparticles, however higher genotoxicity of the uncapped nanoparticles was observed towards the cell lines. Regarding the effects on nontarget microorganisms, in the minimal inhibitory concentration assay only the capped nanoparticles inhibited microorganisms of agricultural importance, while in the molecular analysis of the soil microbiota there were major changes in the soils exposed to the uncapped nanoparticles.

**Conclusions:**

The results suggest that the capping played an important role in controlling nanoparticle size and contributed to the biological activity of the nanoparticles against *S. sclerotiorum*. This study opens perspectives for investigations concerning the application of these nanoparticles for the control of phytopathogens. 
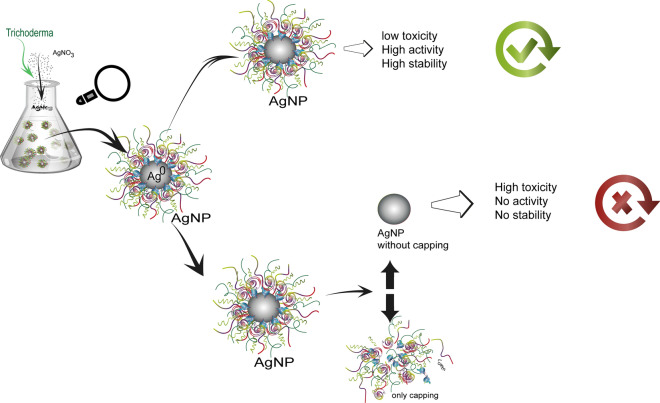

## Background

The use of nanotechnology in the agricultural sector is increasing, making important contributions to improvements in areas including pest control. Among the new nanomaterials, silver nanoparticles (AgNPs) can be highlighted for the control of phytopathogenic microorganisms [1]. These nanoparticles can be synthesized using biological reducing agents and stabilizers, including fungi, bacteria, plants, and algae [2], involving action of the nitrate reductase enzyme and other metabolites [3]. Fungi can be considered as nanobiofactories for the fast and environmentally friendly synthesis of silver nanoparticles [1]. The nanoparticles are formed as a result of the interaction of a metallic precursor with enzymes, coenzymes, and other active substances derived from the organisms, with the resulting nanoparticles being capped with bioactive material [4, 5].

Metallic nanoparticles produced by biogenic synthesis generally possess this capping, which provides stability, while other substances can be added to provide specific activities that potentiate the effects of the nanoparticles [6, 7]. The capping of biogenic nanoparticles is composed of biomolecules and compounds derived from the metabolism of the fungus or other biological agent, such as extracellular proteins, enzymes, amino acids, and secondary metabolites [8, 9]. In the case of nanoparticles produced by chemical synthesis, it is necessary to add capping agents such as surfactants, proteins, and other biomolecules, in order to achieve electrosteric stability. These agents bind to the surfaces of the nanoparticles by means of covalent bonds or chemical interactions, and are not readily degraded [10–12].

Depending on its composition, the capping can also improve nanoparticle biocompatibility, providing an active surface for interaction with biological compounds and conjugation with substances such as medicines, antimicrobials, genetic material, and amino acids [9]. Bhunia et al. reported that silver nanoparticles capped with protein such as human hemoglobin presented greater biocompatibility, compared to uncapped nanoparticles [13]. These characteristics make biogenic nanoparticles more suitable for applications in the areas of health, agriculture, and the environment [14, 15]. However, an important consideration is that the surface capping may influence parameters such as the morphology, aggregation, and dissolution rate of the particles, in addition to affecting cytotoxicity [15]. Hence, before using these nanoparticles in the areas of health and agriculture, it is necessary to determine their physico-chemical characteristics and perform toxicity evaluations on cell lines and nontarget organisms due to their consequent realease into the environment [16].

White mold, a disease affecting more than 450 species of agricultural crops, is caused by the fungus *Sclerotinia sclerotiorum*, considered one of the most important phytopathogens worldwide. This fungus is able to form highly resistant structures (sclerotia) that can remain viable for years in the soil [17]. The control of this disease is based on the application of chemical fungicides and biological control agents, while some studies have reported the inhibitory effects of silver nanoparticles against phytopathogenic fungi [18–23]. In a previous study by our research group, silver nanoparticles were synthesized using the fungus *Trichoderma harzianum*, with and without stimulation of enzyme production, and were found to inhibit *S. sclerotiorum* mycelial growth and sclerotia germination in vitro [23].

Given the importance of the capping on nanoparticles, the aim of the present study was to compare the physico-chemical characteristics of capped and uncapped silver nanoparticles synthesized in our previous study [23], investigate the cappings using infrared spectroscopy, protein analyses, and enzymatic assays, and evaluate the biological activity of the nanoparticles against *S. sclerotiorum*, as well as their toxicity towards different cell lines and nontarget microorganisms.

## Materials and methods

### Materials

This study employed silver nanoparticles previously synthesized using the filtrate of *Trichoderma harzianum*, with enzyme production stimulated by the presence of the cell wall of *Sclerotinia sclerotiorum*, denoted AgNP-TSC (57.02 ± 1.75 nm; −18.70 ± 3.01 mV; polydispersity 0.49 ± 0.01), and nanoparticles produced without stimulation, denoted AgNP-TC (81.84 ± 0.67 nm; −18.30 ± 1.73 mV; polydispersity 0.52 ± 0.00) [23].

The cell lines V79 (Chinese hamster pulmonary fibroblast), 3T3 (albino Swiss mouse embryo fibroblast), and HaCat (human keratinocyte) were obtained from the Rio de Janeiro Cell Bank (Rio de Janeiro, Brazil). The agriculturally important microorganisms *Bradyrhizobium japonicum*, *Pseudomonas aeruginosa*, *Bacillus thuringiensis*, *Beauveria bassiana*, and the phytopathogen *Sclerotinia sclerotiorum* were donated. Thiazolyl Blue Tetrazolium Bromide (MTT) and resazurin were obtained from Sigma-Aldrich. Potato dextrose agar was purchased from Kasvi. Blueye Prestained protein molecular weight marker, the Apoptosis Annexin V AlexaFluor 488 and propidium iodide kit, the Qubit dsDNA HS DNA quantification kit, and SYBR Green were obtained from Invitrogen. Visking MWCO 12–14 kDa dialysis membranes were purchased from Serva. The PowerSoil kit for DNA extraction from soil was obtained from QIAGEN.

### Removal of the nanoparticle cappings

In order to investigate possible differences between capped and uncapped nanoparticles, the capping was removed from half the volume of the samples, as described by Jain et al. [24]. The nanoparticle dispersions were centrifuged, the pellets were resuspended and boiled in 1% sodium dodecyl sulfate (SDS), and a further centrifugation was performed. The supernatants containing the cappings were stored at **−**20 ºC, prior to subsequent protein and enzyme analyses. Nanoparticles without cappings were obtained by boiling the pellets in 60 mM Tris-HCl pH 6.8, followed by dialysis using a Visking MWCO 12–14 kDa membrane. The procedure used to remove the capping from the nanoparticles resulted in two new uncapped samples, giving a total of four samples (two capped and two uncapped). The samples were labeled as follows: AgNP-TSC (capped silver nanoparticles, with stimulation), AgNP-TC (capped silver nanoparticles, without stimulation), AgNP-TS (uncapped silver nanoparticles, with stimulation), and AgNP-T (uncapped silver nanoparticles, without stimulation).

#### Characterization and stability evaluation of the biogenic nanoparticles

After removal of the nanoparticle cappings, the capped and uncapped nanoparticles and the corresponding *T. harzianum* filtrates were analyzed using UV-visible spectroscopy in the wavelength range 200–800 nm, with resolution of 1 nm, using a Shimadzu Multispec 1501 spectrophotometer. Measurements of the pH of the nanoparticles and the filtrates were performed immediately after capping removal at ambient temperature using a pH meter (HMMPB-210).

The techniques of dynamic light scattering (DLS) and microelectrophoresis were used to determine the hydrodynamic diameter, polydispersity, and zeta potential of the samples, employing a ZetaSizer Nano ZS90 analyzer (Malvern Instruments). The readings were made in triplicate, at 25 ºC, with a fixed angle of 90º. The stability of the nanoparticles was evaluated by repeating these analyses six and twelve months after the synthesis. The nanoparticle concentrations were obtained by nanoparticle tracking analysis (NTA), using a NanoSight LM 10 cell and NanoSight v. 2.3 software. The nanoparticles were dispersed in water to a standard working concentration of 1 × 10^10^ NPs.mL^− 1^.

#### Morphological analysis of the nanoparticles

The morphologies of the capped and uncapped nanoparticles were investigated using atomic force microscopy (AFM). Aliquots of the nanoparticles were diluted in ultrapure water and 10 µL volumes were dripped onto silicon AFM plates, followed by keeping in a desiccator until completely dry. The analyses were performed using an easyScan 2 atomic force microscope (Nanosurf, Switzerland) equipped with TapAl-G cantilevers (BudgetSensors, Bulgaria) and operated in noncontact mode at a scan rate of 90 Hz. The micrographs were interpreted using Gwyddion software.

#### Characteristics of the nanoparticle cappings

Fourier transform infrared spectroscopy (FTIR) analyses of the capped and uncapped nanoparticles were performed using a JASCO FT/IR-410 spectrometer. For this, KBr tablets were prepared using a proportion of 1.5% of the solid nanoparticles obtained by freeze-drying of aqueous dispersions. The spectra were acquired in the range from 4000 to 400 cm^− 1^, at 8 cm^− 1^ resolution, with 32 scans.

## Analysis of proteins in the nanoparticle cappings

The sodium dodecyl sulfate polyacrylamide gel electrophoresis (SDS-PAGE) was used to investigate the presence of *T. harzianum* proteins in the filtrates used for the synthesis and in the cappings removed from the nanoparticles, as well as in the capped and uncapped nanoparticles. The assay was performed based on the methodology from Chowdhury et al. with some adaptations [25]. The samples were mixed with buffer (1:1 v:v ratio), heated at 95 ºC for 10 min, centrifuged at 14,000 rpm for 1 min, and loaded onto a 12% SDS-Polyacrilamide gel in the following order: (1) Blueye Prestained ladder (Invitrogen) 11–245 kDa molecular weight marker; (2) Filtrate from *T. harzianum* without stimulation; (3) Capping without stimulation; (4) AgNP-TC; (5) AgNP-T; (6) Filtrate of *T. harzianum* with stimulation; (7) Capping with stimulation; (8) AgNP-TSC; (9) AgNP-TS. Electrophoresis was performed at 200 V and 20 mA, until the dye reached the lower region of the gel. The gel was stained using ammoniacal silver solution and analysis of the protein profiles was performed visually, based on the molecular weight marker.

### **Specific activity of the*Trichoderma harzianum*hydrolytic enzymes**

The protein concentrations in the filtrates, cappings, and nanoparticles AgNP-TSC, AgNP-TC, AgNP-TS and AgNP-T were determined using Bradford’s reagent and bovine serum albumin (1, 0.5, 0.25, and 0.125 mg.mL^− 1^) as standard [26]. Evaluation of the specific activities of the *T. harzianum* hydrolytic enzymes N-acetylglucosaminidase (NAGase), β-1,3-glucanase, chitinase, and acid protease in the filtrates, cappings, and nanoparticles was performed based on the methodology described by Qualhato et al. [27]. The assays employed 96-well microplates and the following samples: *T. harzianum* filtrate without stimulation; capping without stimulation; AgNP-TC; AgNP-T; *T. harzianum* filtrate with stimulation; capping with stimulation; AgNP-TSC; AgNP-TS.

### **Biological activity of the nanoparticles towards the phytopathogen*Sclerotinia sclerotiorum***

The activity of the capped and uncapped nanoparticles for the control of *S. sclerotiorum* was evaluated using assays of the growth of the phytopathogen on potato dextrose agar supplemented with the samples at a concentration of 3 × 10^9^ NPs.mL^− 1^ [19, 23]. After 15 days of culture, the mycelium growth halos were measured and the numbers of new sclerotia were counted.

### **Cytotoxic and genotoxic potentials of the nanoparticles towards cell lines and*Allium cepa***

Cytotoxicity and genotoxicity analyses were performed to compare the effects of the capped nanoparticles (AgNP-TSC and AgNP-TC) and the corresponding uncapped nanoparticles (AgNP-TS and AgNP-T).

#### Cytotoxicity evaluation

The cytotoxicity of the samples was evaluated using the V79, 3T3, and HaCat cell lines. Assays of mitochondrial activity employed the Thiazolyl Blue Tetrazolium Bromide (MTT). Cell viability, apoptosis, and necrosis were determined by imaging cytometry. Cell viability was evaluated using the trypan blue dye exclusion method.

For the MTT assay, the cells were plated (5 × 10^4^ cells/well) and exposed to the nanoparticles at concentrations between 0.1 × 10^9^ and 3.5 × 10^9^ NPs.mL^− 1^ for 24 h. The MTT solution (5 mg.mL^− 1^) was added, followed by incubation for 3 h, fixation of the cells with DMSO, and absorbance reading at 540 nm. In the analyses using imaging cytometry and trypan blue exclusion, the cells were exposed for 1 h to the nanoparticles at 3 × 10^9^ NPs.mL^− 1^. The imaging cytometry analyses of cell viability, apoptosis, and necrosis were performed using the Annexin V AlexaFluor® 488 and propidium iodide kit (Invitrogen), according to the manufacturer’s instructions, with the readings obtained using a Tali™ Image Cytometer. In the trypan blue exclusion assay, immediately after the end of exposure to the nanoparticles at 3 × 10^9^ NPs.mL^− 1^, the cells were stained with trypan blue, followed by counting using an optical microscope, in triplicate, considering cells stained blue to be dead.

### Genotoxicity evaluation

The genotoxicity of the nanoparticles was determined using *Allium cepa* and comet assays. The *Allium cepa* assay was performed as described by Lima et al. [28]. The exposure of *Allium cepa* roots to the nanoparticle samples was performed for 24 h, using concentrations of 1 × 10^10^ and 3 × 10^9^ NPs.mL^− 1^, followed by fixation and hydrolysis of the roots, preparation of slides (in triplicate), and analysis by optical microscopy. Out of the total number of cells, counting was made of those that presented cell division, giving the mitotic index (MI), and of these, those that presented chromosomal alterations, giving the alteration index (AI).

Comet assay was performed according to the methodology described by Singh et al. [29], with adaptations. The same cell lines used in the cytotoxicity assays were exposed for 1 h to the samples at 3 × 10^9^ NPs.mL^− 1^, followed by preparation of the slides, immersion in lysis solution for 1 h, neutralization, immersion in electrophoresis buffer for 20 min, and electrophoresis for 20 min at 22 V and 300 mA. The slides were then fixed, stained, and evaluated under a microscope, with visual scoring [30].

### Toxicity of the nanoparticles towards nontarget microorganisms

#### Evaluation of minimum inhibitory concentration (MIC) towards microorganisms of agricultural importance

MIC assay was performed using the agriculturally important microorganisms *Bradyrhizobium japonicum*, *Pseudomonas aeruginosa*, *Bacillus thuringiensis*, and *Beauveria bassiana*. The microorganisms were cultured for 24 h, transferred to 96-well plates at a concentration of 5 × 10^5^ CFU.mL^− 1^, and exposed to the capped and uncapped nanoparticles at decreasing concentrations between 4.0 × 10^9^ and 1.0 × 10^8^ NPs.mL^− 1^. After incubation for 24 h at 37 ºC, resazurin solution (6.75 mg.mL^− 1^) was added and the plates were incubated for a further 24 h, followed by visual color assessment.

#### Molecular qPCR analyses of the effects of the nanoparticles on soil microbiota

Investigation was made of possible effects of the nanoparticles on soil bacteria that participate in the nitrogen cycle during the processes of fixation and denitrification. For this, the soil was exposed to the nanoparticles at a concentration of 3 × 10^9^ NPs.mL^− 1^, in microcosms containing 10 g of soil [31]. A control was also prepared using ultrapure water. The microcosms were kept in the dark at ambient temperature. On the day of exposure, DNA was extracted from an untreated soil sample (denoted soil zero), in order to obtain the initial conditions. Extractions of DNA from all the samples were then performed 15, 90, 180, and 360 days after exposure, using the PowerSoil DNA Isolation Kit (QIAGEN), followed by DNA quantification using the Qubit dsDNA HS kit, with a Qubit 3.0 fluorometer, and dilution to a final concentration of 100 ng mL^− 1^. Quantification of the genes of the nitrogen cycle bacteria was performed with specific primers, using the real-time polymerase chain reaction (qPCR) with Syber Green [32]. Relative quantification of the DNA was performed using the 16s rRNA gene as a reference. All the samples were analyzed in triplicate.

### Statistical analyses

Statistical analyses employed one-way analysis of variance (ANOVA) followed by Tukey’s test (significance level of p < 0.05), performed using GraphPad Prism 7.0 software.

## Results and discussion

### Characterization and stability of the biogenic nanoparticles

**Fig. 1:**
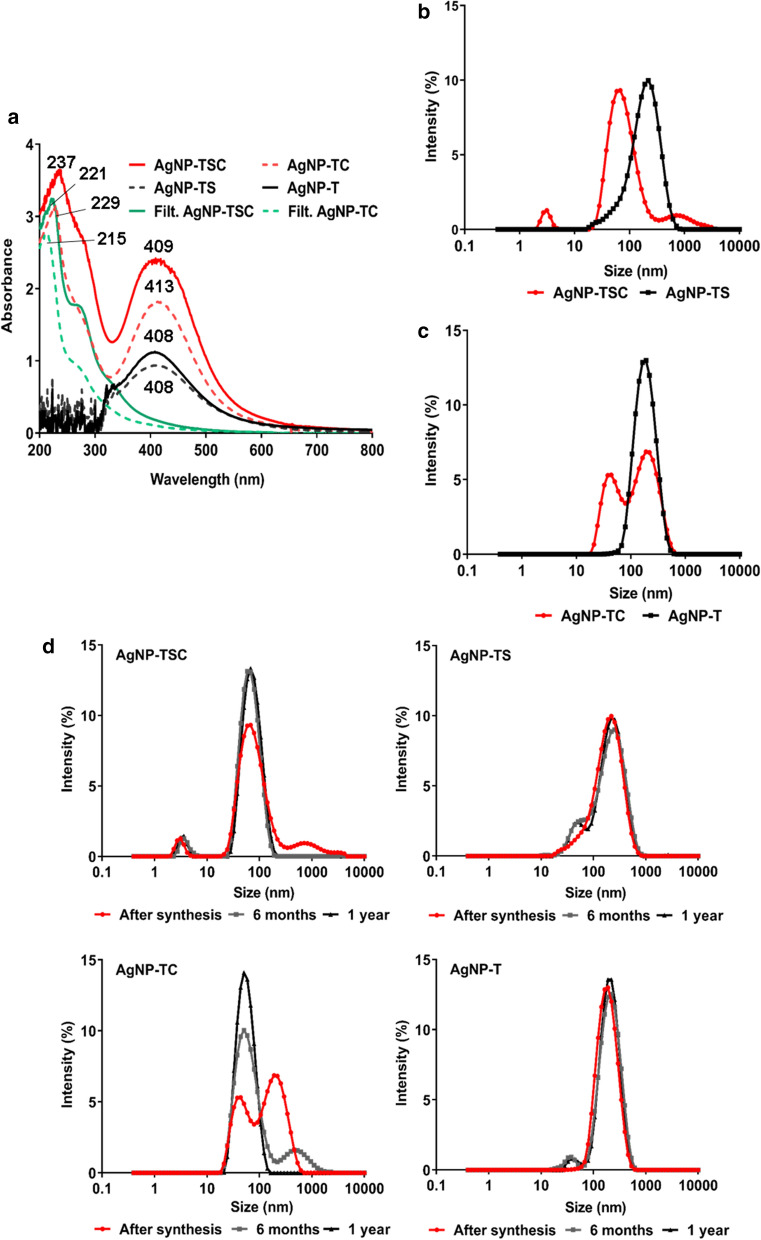
Physico-chemical characterization of the nanoparticles. **a** UV-Vis spectra of the nanoparticles and the corresponding filtrates. **b** and **c** Hydrodynamic diameter distributions obtained by DLS. **d**: Stability evaluation of AgNP-TSC (capped, with stimulation), AgNP-TC (capped, without stimulation), AgNP-TS (uncapped, with stimulation), and AgNP-T (uncapped, without stimulation)

The UV-Vis analyses of all the samples showed absorbance peaks between 400 and 420 nm, characteristic of elemental silver (Ag^0^). The spectra for the capped nanoparticle samples AgNP-TSC and AgNP-TC showed peaks between 200 and 300 nm, which were similar to peaks in the corresponding filtrates and indicated the presence of organic compounds such as amino acid residues, secondary metabolites, and proteins. In the same region, noise signals were observed for the AgNP-TS and AgNP-T samples, which could be attributed to the process of removing the capping and eliminating the biomolecules (Fig. 1a).

The nanoparticles presented different hydrodynamic diameter distributions, which could be attributed to the synthesis conditions employed, especially the presence or absence of a capping. The AgNP-TSC sample showed peaks at 3 nm (6.0%), 59 nm (88.3%), and 825 nm (5.6%) (Fig. 1b), while the AgNP-TC sample showed peaks at 32 nm (39.3%) and 190 nm (60.7%) (Fig. 1c). The formation of particle populations with different sizes for nanoparticles synthesized using biogenic routes has been observed previously [33, 34]. After removal of the cappings, these nanoparticles showed size distribution peaks at 220 nm (100%) for AgNP-TS (Fig. 1c) and at 190 nm (100%) for AgNP-T (Fig. 1c). Increase of the particle diameter after removal of the capping was probably due to subsequent aggregation of the nanoparticles [35].

Polydispersity index values of 0.49, 0.52, 0.42, and 0.25 were obtained for the AgNP-TSC, AgNP-TC, AgNP-TS, and AgNP-T samples, respectively. The lowest value for the AgNP-T sample reflected the smallest variation of nanoparticle size, although the larger diameter of these particles was indicative of aggregation followed by stabilization. In the case of the zeta potential, corresponding to the charge formed at the interface between the nanoparticles and the dispersion medium, all the samples presented negative values (−16.7, −16.3, −33.3, and − 15.2 mV for AgNP-TSC, AgNP-TC, AgNP-TS, and AgNP-T, respectively), with the AgNP-TS sample showing the highest electronegativity. Although a higher zeta potential generally indicates higher nanoparticle stability, the stability of biogenic nanoparticles is mainly influenced by the capping of biomolecules [12]. Different cappings produce different surface charges, which can influence the activity of the nanoparticles and their cytotoxicity [36, 37].

The stability of the nanoparticles was evaluated by DLS analyses performed 6 and 12 months after their synthesis (Fig. 1d). In most cases, no changes in nanoparticle hydrodynamic diameter were observed. An exception was the AgNP-TC sample, which initially presented a bimodal particle size distribution, while after 6 months there was a shift towards smaller diameter nanoparticles. After one year, there was the emergence of a monomodal distribution, with the nanoparticles presenting a smaller diameter than observed at the start of the experiment. A possible explanation is that soon after synthesis residues of *T. harzianum* remain in the colloidal solution of nanoparticles, which can cause them to agglomerate and present larger diameter populations through DLS technique. Over time these organic compounds of the fungus are degraded and the capped nanoparticles are dispersed from the agglomerates, presenting a monomodal distribution, that is, a single population of smaller diameter. Despite this change in diameter, both the capped nanoparticles continued to exhibit biological activity for the control of *S. sclerotiorum* after 12 months, with no evidence of color change, flocculation, or sedimentation. The maintenance of the activity could be attributed to the fungal biomolecules surrounding the nanoparticles, which provided steric stability [38]. The stability of nanoparticles in a dispersion is essential for ensuring their biological activity [39]. Also, regarding stability of biogenic nanoparticles it is important to emphasize that there are other factors which could be involved in the stability over time, in special the zeta potential of the particles as stated above.

The pH values of the dispersions of both types of capped nanoparticles were close to the values for the corresponding filtrates (pH 7.2–7.3). Lower values were obtained for the uncapped nanoparticles (pH 4.9–5.0), which was probably because the nanoparticles were not capped by compounds derived from the filtrate. During the synthesis, OH^−^ ions supply electrons for the reduction of silver ions and are adsorbed on the surfaces of the nanoparticles, ensuring their stability and avoiding size changes, resulting in a more alkaline dispersion [14]. When the capping is removed, these OH^−^ ions are lost, changing the pH of the dispersion and exposing the Ag^0^ nanoparticles to the aqueous solution, initiating an ionization process and consequent loss of stability [40]. Uncapped nanoparticles are more readily ionized in aqueous dispersions, with lower pH of the dispersion favoring their dissolution [24, 41].

### Morphological analysis of the nanoparticles

**Fig. 2:**
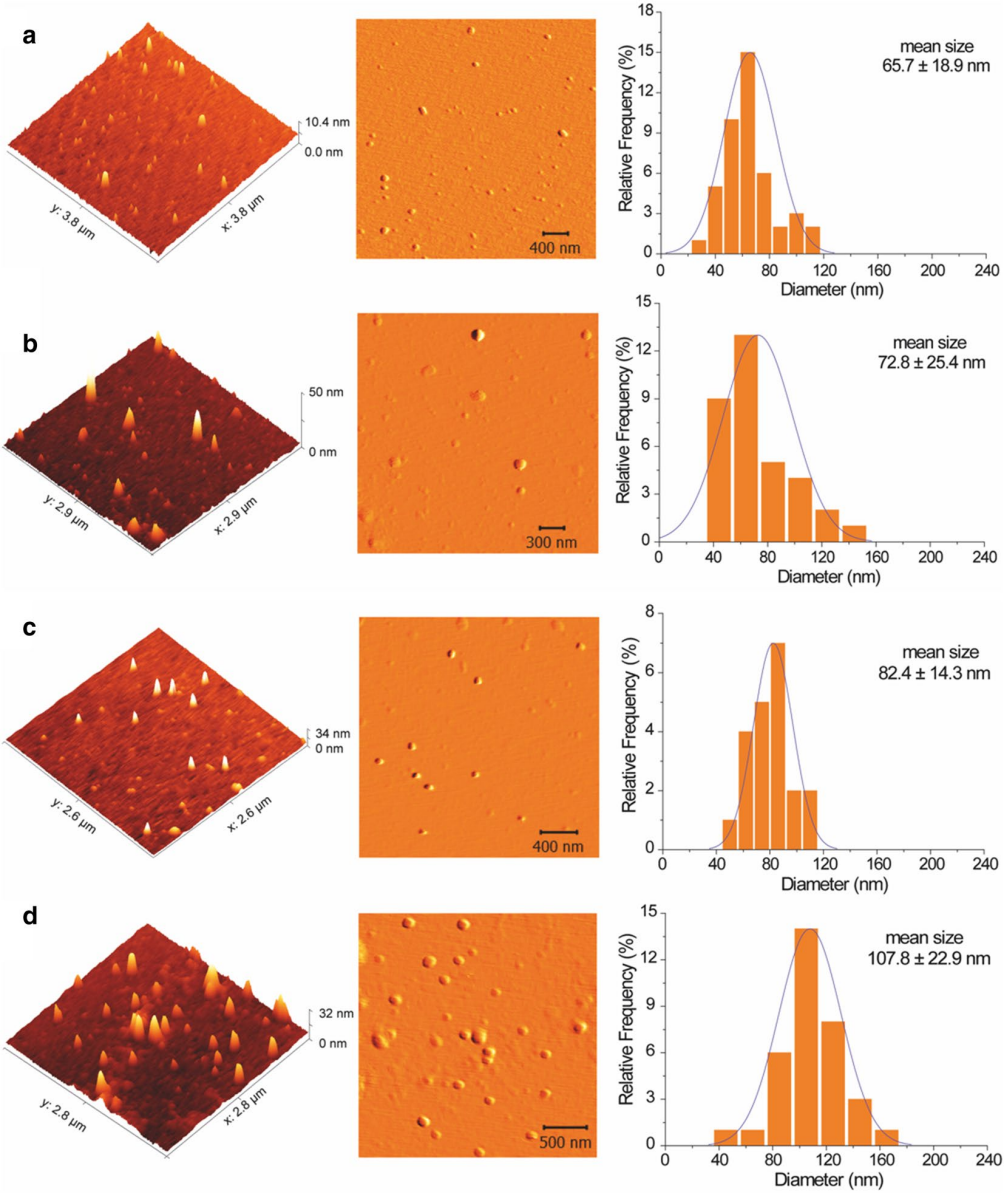
Atomic force microscopy (AFM) images and size distributions of the capped nanoparticles AgNP-TSC (**a**) and AgNP-TC (**b**) and the uncapped nanoparticles AgNP-TS (**c**) and AgNP-T (**d**)

The use of atomic force microscopy showed that the four nanoparticle samples presented spherical morphology, while the uncapped nanoparticles had larger mean diameters (Fig. 2), in agreement with the diameter distributions obtained by DLS analysis. Previous work has found that the morphology and size of nanoparticles are directly influenced by the synthesis conditions [42].

### Characterization of the nanoparticle cappings

FTIR is an important tool for characterization of systems such as those produced here, enabling the identification of specific characteristics of the nanoparticles and their precursors. For example, the detection of proteins responsible for the biogenic synthesis and stability of silver nanoparticles has been reported in previous studies [25, 43, 44]. The interactions of nanoparticles with these proteins and amino acid residues can occur by means of covalent bonds to amino groups and cysteine residues, as well as by electrostatic connections involving carboxyl groups [38, 44]. Daphedar and Taranath used FTIR to detect bands of protein groups in the spectra for nanoparticles produced by biogenic synthesis and reported that the phytochemical components of the extract used in the synthesis (proteins, carboxylic acids, flavonoids, alcohols, and phenols) acted in the processes of reduction, capping formation, and stabilization [45].

**Fig. 3:**
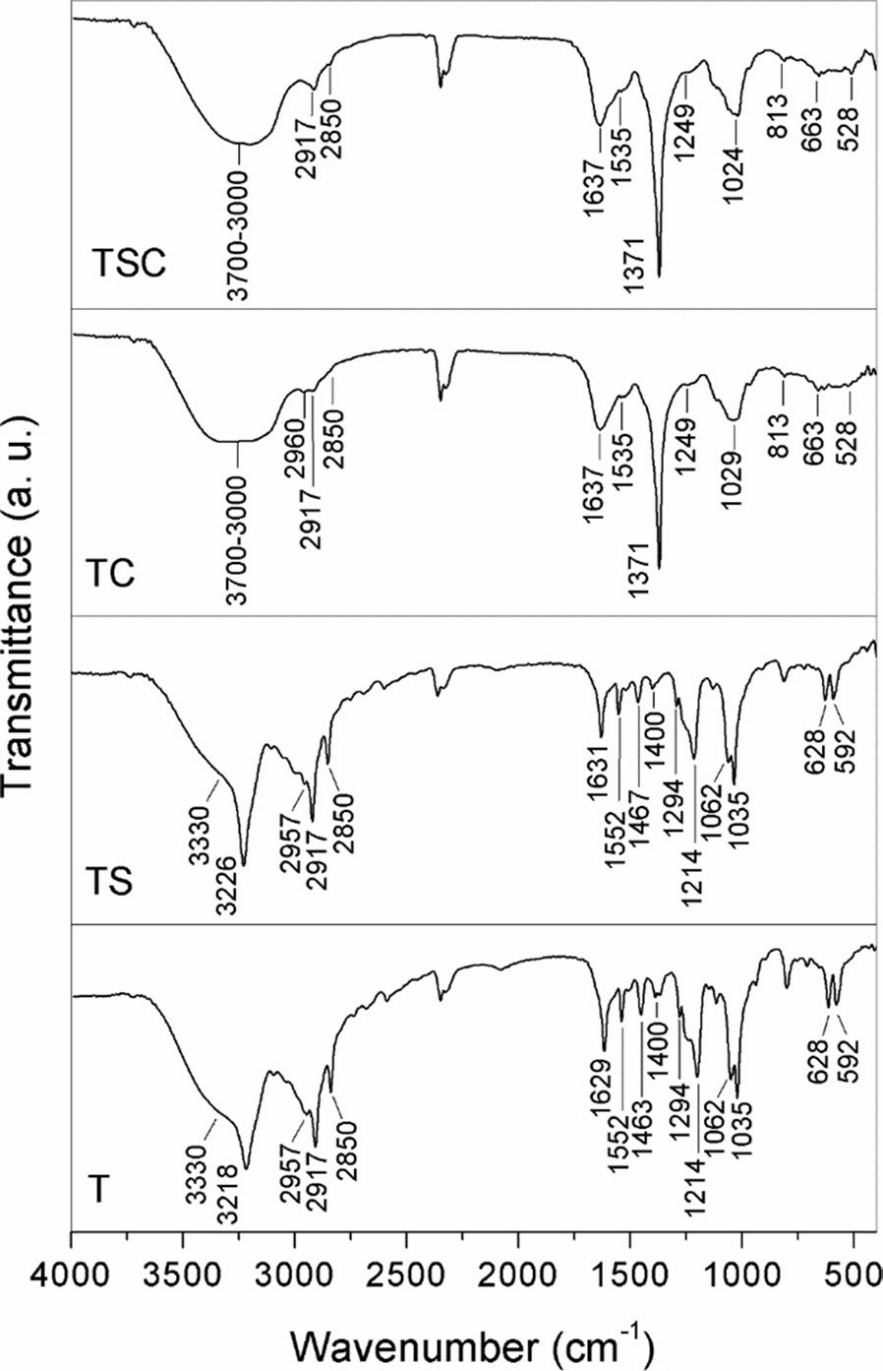
FTIR spectra of the capped (AgNP-TSC and AgNP-TC) and uncapped nanoparticles (AgNP-TS and AgNP-T)

In the present work, the infrared spectra for the capped nanoparticles (TSC and TC) presented bands characteristic of functional groups ascribed to the active fungal biomolecules (Fig. 3). A broad band at 3700−3000 cm^− 1^ could be attributed to O–H stretching vibrations of hydroxyl groups [46, 47]. A band at 2960 cm^− 1^ was due to stretching of the amide NH groups [46] in the structures of proteins and hydrolytic enzymes such as glucanases and chitinases. Bands at 2917 and 2850 cm^− 1^ were characteristic of C–H stretching [8, 48], while bands at 1637 and 1535 cm^− 1^ could be attributed to amides I and II, respectively [38, 47]. An intense band at 1371 cm^− 1^ was attributed to bending vibration of C–H of methyl groups [46, 49] and/or stretching of C–N of aromatic amines [8, 38, 47]. A low intensity band at 1249 cm^− 1^ corresponded to amine C–N stretching [47, 48]. Absorption at around 1024 cm^− 1^ was attributed to ether group C–O stretching [46, 47, 50]. The presence of these functional groups in the capped nanoparticles indicated that the capping was formed by structures derived from the fungus, such as proteins, hydrolytic enzymes, and amino acid residues produced by enzymatic proteolysis.

The features observed in the absorption spectra were in agreement with previous results reported in the literature. Gurunathan et al. synthesized silver nanoparticles using filtrates of the bacterium *Bacillus tequilensis* and the fungus *Calocybe indica*, with the spectral bands for both nanoparticles being close to those found for the capped nanoparticles in the present study [36]. Jain et al. obtained similar results for silver nanoparticles synthesized using filtrate of the fungus *Aspergillus flavus* NJP08. In both studies, formation of a capping around the nanoparticles was attributed to the high capacity of C–O groups of amino acid residues to bind with metals, where the stability of the nanoparticles was due to the interactions with proteins [44].

The characteristic bands of the capped nanoparticles were not observed for the nanoparticles that had undergone the process of capping removal. These nanoparticles presented bands corresponding to the Tris-HCl buffer in which the nanoparticles were resuspended [51, 52]. A broad band at 3300 cm^− 1^ corresponded to O–H stretching, while a band at around 3226 cm^− 1^ could be attributed to symmetrical N–H stretching. Bands in the region between 2957 and 2850 cm^− 1^ were ascribed to symmetric and asymmetric stretching vibrations of CH_2_. Bands at 1629 and 1552 cm^− 1^ were characteristic of in-plane and out-of-plane symmetric angular deformation of NH_2_. Bands at 1463 and 1400 cm^− 1^ corresponded to CH_2_ deformation and C–C vibration, respectively. Bands at 1294 and 1214 cm^− 1^ were attributed to the deformations of OH, while signals at 1062 and 1035 cm^− 1^ were due to deformations of C–O. Finally, a doublet at 628 and 592 cm^− 1^ corresponded to C–C–C deformation. These results were in agreement with the work by Jain et al. who removed the capping from silver nanoparticles synthesized using the filtrate of *Aspergillus* sp. NJP02 [24]. The profile of the FTIR spectrum changed, with the vibrational bands corresponding to amide I, amide II, and aliphatic amine C–N stretching disappearing, hence confirming removal of the capping.

### Analysis of proteins in the nanoparticle cappings

**Fig. 4:**
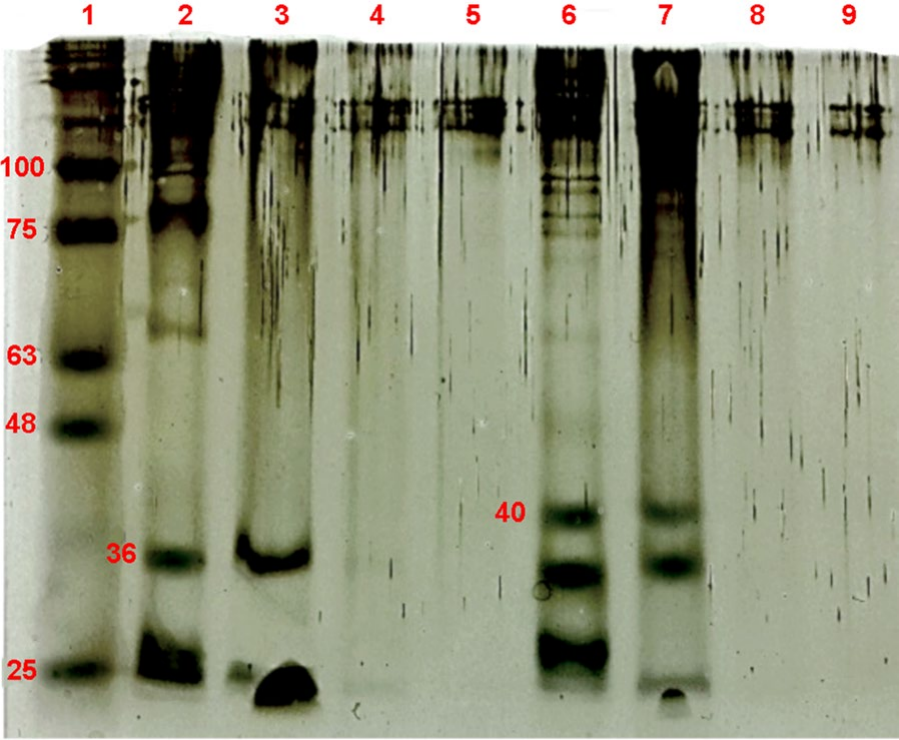
SDS-PAGE analysis of the nanoparticles and the corresponding filtrates and cappings: **1**. Molecular weight marker, 11–245 kDa (Blueye Prestained ladder, Invitrogen); (**2**) Filtrate of *T. harzianum* without stimulation; (**3**) Capping without stimulation; (**4**) AgNP-TC; (**5**) AgNP-T; (**6**) Filtrate of *T. harzianum* with stimulation; (**7**) Capping with stimulation; (**8**) AgNP-TSC; (**9**) AgNP-TS

SDS-PAGE assay was performed in order to confirm that the proteins in the filtrate used for synthesis of the nanoparticles were retained in the cappings. The protein profiles of both filtrates exhibited bands that were also shown by the corresponding cappings, indicating that the filtrate proteins were also present in the layer surrounding the nanoparticles (Fig. 4).

The filtrate and capping samples showed bands at 36 kDa and 40 kDa, corresponding to the molecular weights of the *T. harzianum* β-1,3-glucanase and chitinase enzymes, respectively, indicating the presence of these enzymes, which was confirmed by specific enzymatic activity analyses [53, 54]. No clear bands were observed for the capped and uncapped nanoparticle samples. In the case of the capped nanoparticles, the strong interactions between the proteins and the nanoparticles prevented the migration of proteins in the gel [44], while in the case of the uncapped nanoparticles, the proteins were eliminated from the samples during the processes of capping removal and dialysis.

The appearance of bands corresponding to the same molecular weight in the analyses of the filtrates and cappings provided confirmation that the filtrate proteins had capped the nanoparticles [44]. Similar results were reported by Rodrigues et al. who obtained the same protein bands (at 75, 122, 191, and 328 kDa) for the filtrate and the capping of nanoparticles synthesized using *Aspergillus tubingensis*, which confirmed the participation of the filtrate proteins in the nanoparticle synthesis process and their retention in the layer surrounding the nanoparticles [43]. Jain et al. used SDS-PAGE to analyze the filtrate of the fungus *Aspergillus flavus*, the nanoparticles synthesized using this fungus, and the capping of the nanoparticles, which was removed by boiling in 1% SDS. The fungus filtrate presented two intense bands at 35 and 32 kDa, while the 35 kDa band was also observed for the nanoparticle capping. The synthesis was suggested to occur in two stages, with the 32 kDa protein firstly reducing the silver ions to form nanoparticles, followed by bonding of the 35 kDa protein to the nanoparticles, making them stable [44]. Chowdhury et al. reported the presence of a band at 85 kDa for the filtrate and the capping of silver nanoparticles obtained using *Macrophomina phaseolina*, indicating the presence of fungal compounds enveloping the nanoparticles. The authors attributed this band to a component of the capping that conferred stability to the nanoparticles [25].

#### **Specific activity of*Trichoderma harzianum*hydrolytic enzymes**

**Fig. 5:**
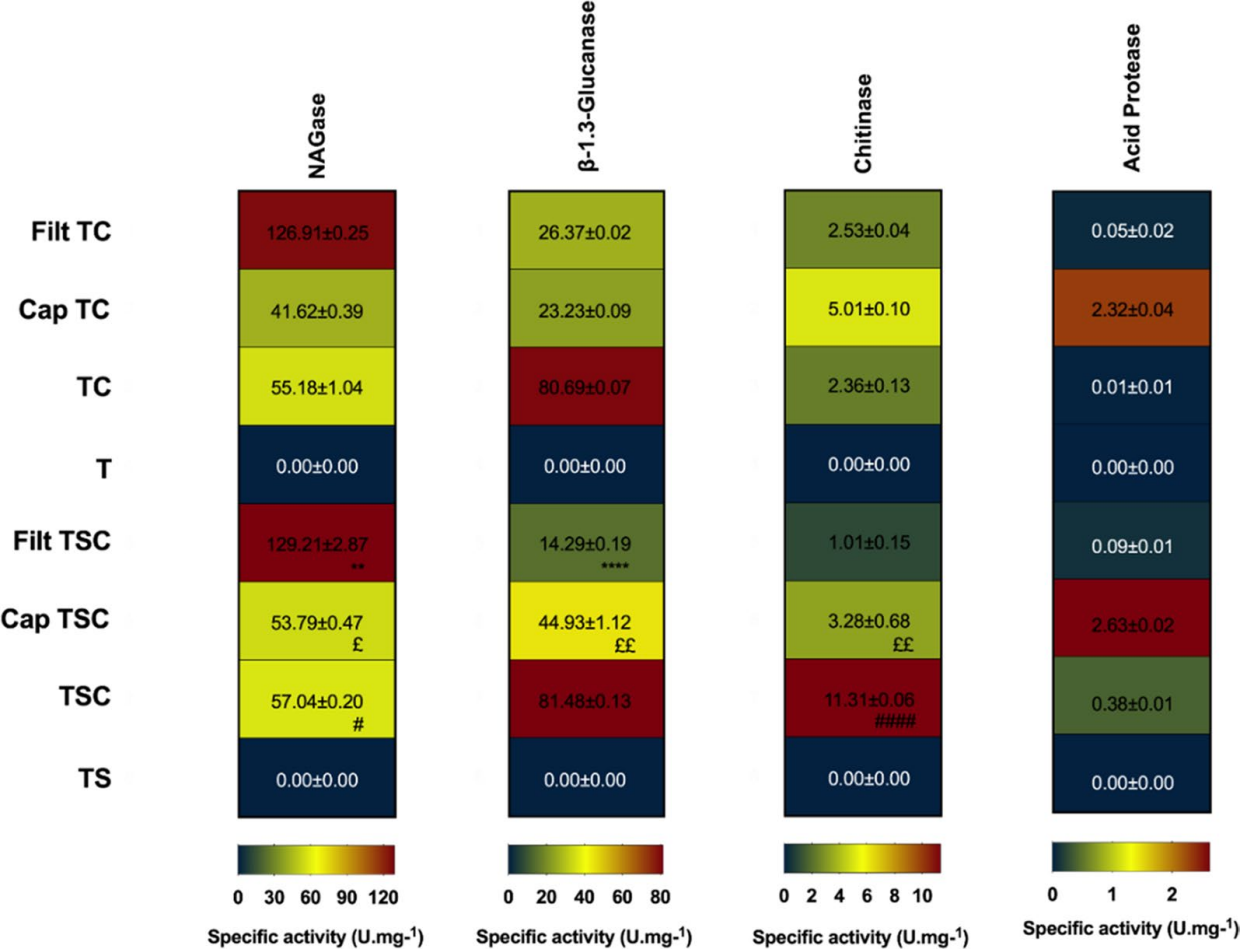
Specific activity (U.mg^− 1^) of the *Trichoderma harzianum* hydrolytic enzymes N-acetylglucosaminidase (NAGase), β-1,3-glucanase, chitinase, and acid protease. Filt TSC: filtrate with stimulation; Cap TSC: capping with stimulation; TSC: capped nanoparticles with stimulation; TS: uncapped nanoparticles with stimulation; Filt TC: filtrate without stimulation; Cap TC: capping without stimulation; TC: capped nanoparticles without stimulation; T: uncapped nanoparticles without stimulation. Statistically significant differences (p < 0.05) are indicated by * for comparison between Filt TC and Filt TSC, £ for comparison between Cap TC and Cap TSC, and # for comparison between TC and TSC. The greater the number of symbols the greater the statistical significance

Determination of the specific activity of *T. harzianum* hydrolytic enzymes that act in the biological control of phytopathogens revealed the presence of these enzymes in the filtrates, cappings, and capped nanoparticles, while their activities were absent in the uncapped nanoparticle samples. Among the enzymes studied, the highest activity was generally observed for NAGase, followed by β-1,3-glucanase, while chitinase and acid protease showed low activities. The specific enzymatic activity profiles of the four enzymes are shown in Fig. 5.

Higher β-1,3-glucanase activity was observed for the capped nanoparticles AgNP-TSC and AgNP-TC, while NAGase showed higher activity in both filtrates. The highest chitinase activity was observed for AgNP-TSC, followed by the capping of AgNP-TC, while the highest protease activity was found for the capping of AgNP-TSC. These differences could be explained by the processes to which the samples were submitted, such as removal of the capping, involving heating and centrifugation steps, which had different effects according to the particular enzymes.

The main finding of these analyses, in agreement with the SDS-PAGE and FTIR results, was that the enzymes from the filtrates remained in the cappings, as well as in the capped nanoparticles. This provided an explanation for the greater inhibitory activities of the capped nanoparticles towards the germination and mycelial growth of *S. sclerotiorum*, compared to the uncapped nanoparticles. It is likely that the enzymes present in the nanoparticle cappings could act synergistically with the nanoparticles, enhancing their effects, although further investigations will be needed to confirm this possibility.

The presence of proteins and enzymes in capped biogenic nanoparticles deserves special attention, given the importance of this capping in terms of the stability, biocompatibility, and possible enhanced biological activity of these nanoparticles.

#### **Biological activity of the nanoparticles towards the phytopathogen*Sclerotinia sclerotiorum***

**Fig. 6:**
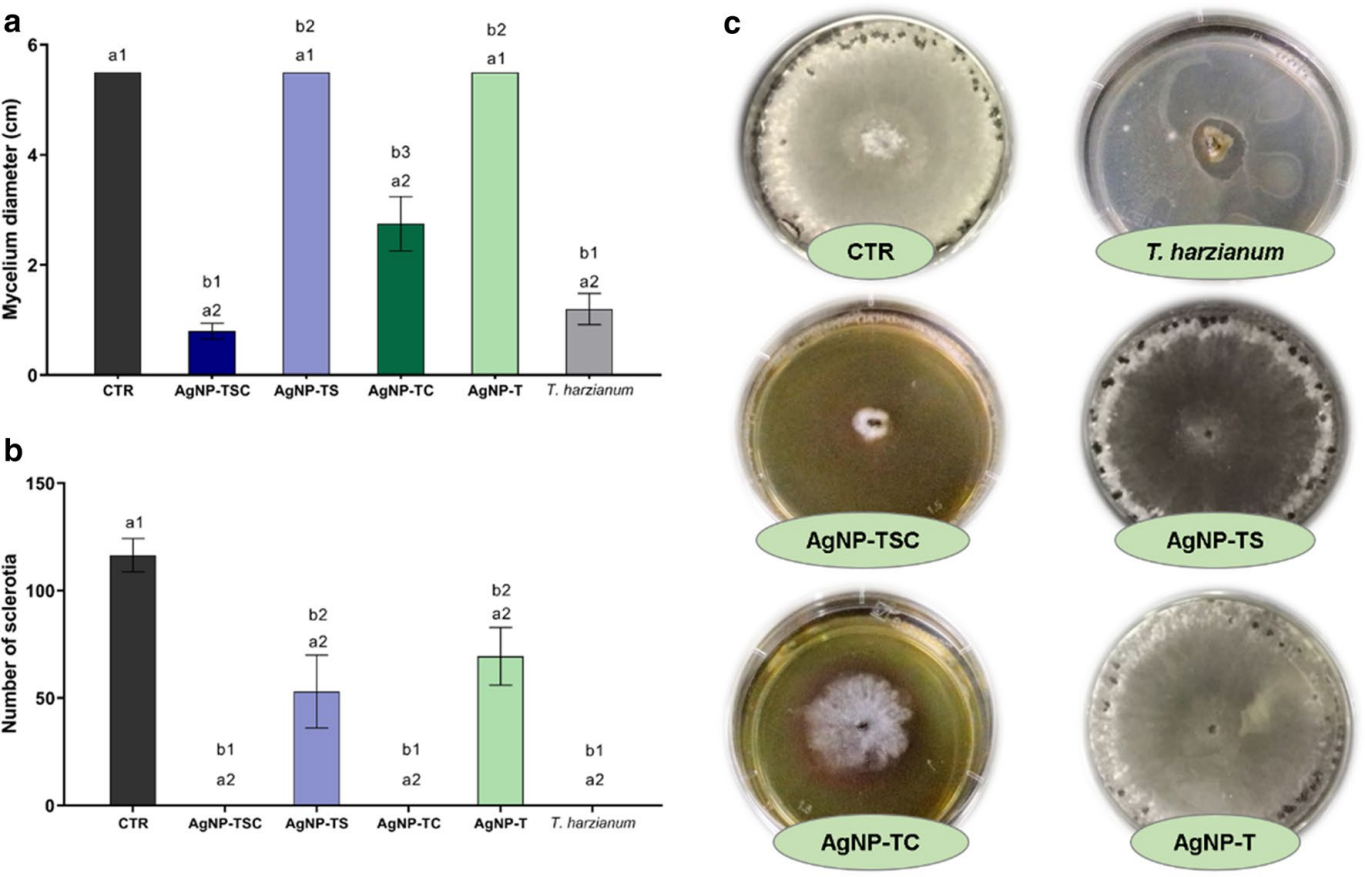
Biological activities of the AgNP-TSC, AgNP-TS, AgNP-TC, and AgNP-T nanoparticles (3 × 10^9^ NPs.mL^− 1^) towards *S. sclerotiorum*. **a** Mycelium diameters; **b** numbers of new sclerotia; **c** visual appearances of the cultures. Statistical analysis: a. control × AgNP; b. AgNP × AgNP. Different numbers indicate statistically significant differences (p < 0.05)

Evaluation was made of the biological activity of the nanoparticles in terms of *S. sclerotiorum* mycelium growth and new sclerotia formation, comparing the effects of the capped and uncapped nanoparticles in culture medium supplemented with the samples. Decreased mycelium growth was observed for the fungus exposed to the capped nanoparticles, for which no formation of new sclerotia was observed, with the best results using AgNP-TSC. In contrast, use of the uncapped nanoparticles resulted in mycelium growth throughout the areas of the plates, equivalent to the control plates, as well as the formation of new sclerotia (Fig. 6a and b). The visual appearances of the cultures are shown in Fig. 6c

The absence of any effect of the uncapped nanoparticles could have been due to their increased size, since larger nanoparticles have been found to present lower cytotoxicity [55, 56]. Another important finding was the presence of active compounds derived from *T. harzianum* in the capped nanoparticles, which could contribute to inhibition of the phytopathogen. Previous studies have found that during the processes of reduction of silver nitrate and binding to the surfaces of the nanoparticles, the proteins do not undergo deformations of the secondary and tertiary structures, or disruption of the covalent bonds [38, 44]. This suggests that the proteins did not suffer from loss of functionality, so they could contribute to the biological activity of the nanoparticles.

Depending on the characteristics and composition of the capping on biogenic nanoparticles, bonds formed with different molecules can result in new functionalities and improvements in colloidal stability [57], as well as mechanisms for the internalization of nanoparticles in cells [58]. Zewde et al. reported that the presence or absence of a capping, the type of capping, and its density could greatly influence the antimicrobial effects and the cytotoxicity potential of nanoparticles [12]. Such findings suggest that the nanoparticle capping could exert antifungal activity, enabling the control of *S. sclerotiorum* and contributing to maintenance of the antifungal properties of the nanoparticles themselves, providing them with greater stability.

Several previous studies have investigated the potential of biogenic nanoparticles for use in the control of phytopathogenic fungi. Elamawi et al. used filtrate of the fungus *Trichoderma longibrachiatum* to synthesize silver nanoparticles that were evaluated in vitro and presented inhibitory effects against the phytopathogenic fungi *Fusarium verticillioides*, *Fusarium moniliforme*, *Penicillium brevicompactum*, *Helminthosporium oryzae*, and *Pyricularia grisea*. FTIR analyses revealed bands corresponding to proteins bound to the surfaces of the nanoparticles, which appeared to contribute to stabilization of the particles and avoidance of agglomeration [39]. Abboud synthesized silver nanoparticles using *Trichoderma harzianum* and evaluated their effects against *Fusarium oxysporum*, *Alternaria alternata*, and *Trichoderma harzianum*. Concentration-dependent inhibition of fungal colony formation was observed [59]. Mishra et al. employed silver nanoparticles synthesized using the bacterium *Stenotrophomonas* sp. as a nanofungicide for the control of foliar phytopathogens such as *Alternaria alternata*, *Curvularia lunata*, and *Bipolaris sorokiniana*, as well as the phytopathogen *Sclerotium rolfsii* in soil. Exposure to the nanoparticles at low concentrations resulted in complete inhibition of the conidia and sclerotia of the foliar phytopathogens [60].

**Fig. 7:**
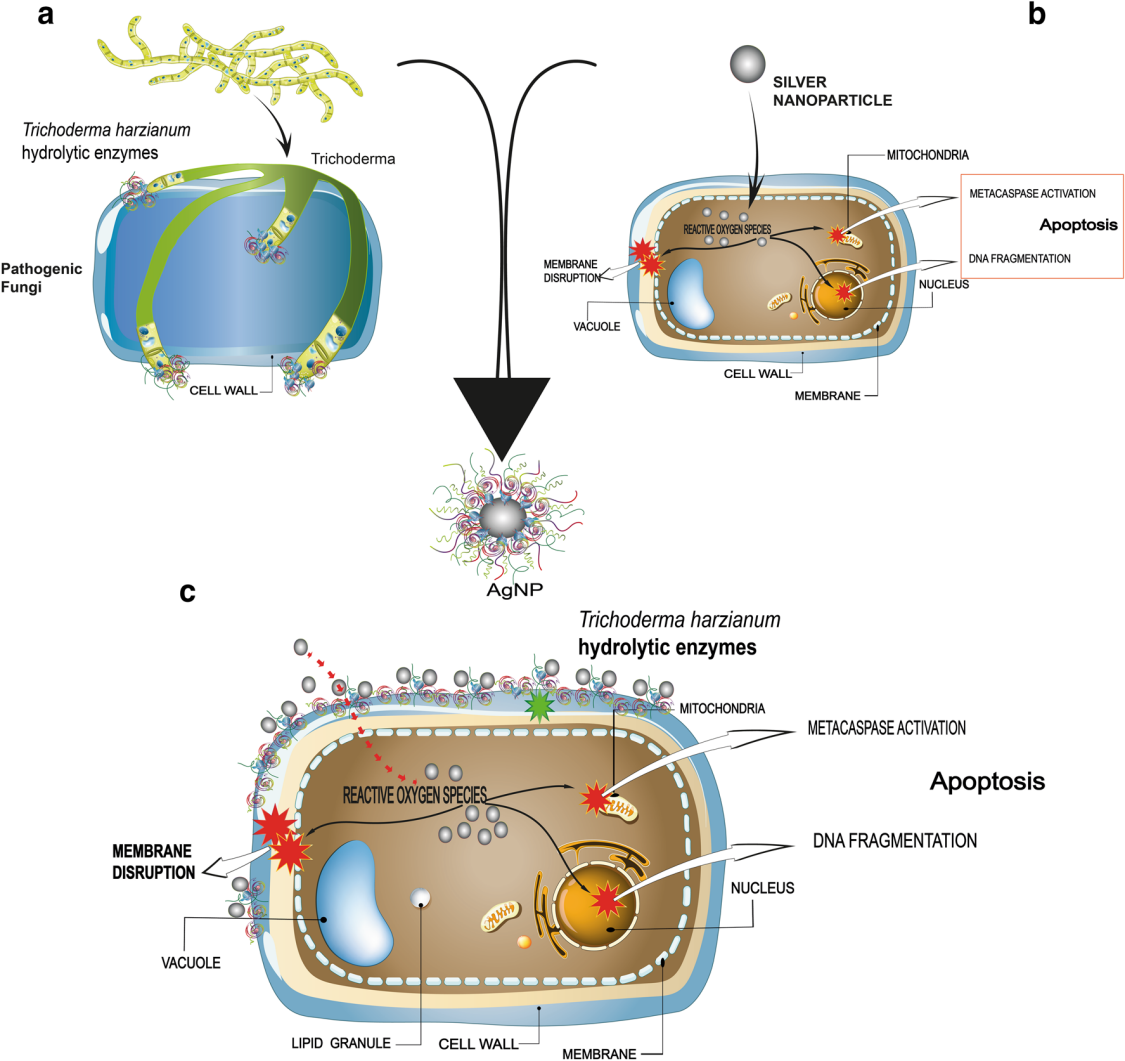
Schematic representation of the possible mechanism of action of the capped silver nanoparticles against *S. sclerotiorum.* **a** degradation of pathogenic fungi cell wall by *T. harzianum* hydrolytic enzymes; **b** membrane disruption and oxidative stress in pathogenic fungi cells caused by silver nanoparticles; **c** synergistic effect of the biogenic silver nanoparticles and the capping hydrolytic enzymes against *S. sclerotiorum*

*Trichoderma harzianum* acts as an antagonist against phytopathogenic microorganisms through direct and indirect mechanisms. Mycoparasitism is a direct mechanism in which, initially, the carbohydrates of *T. harzianum* cell wall are bound to the phytopathogen lectins, followed by the hyphae winding, formation of appresoria and penetration into the target fungus hyphae. After this contact, hydrolytic enzymes such as chitinase and β-1,3-glucanase are released which corrupt the cell wall of the phytopathogen, thus allowing the establishment of parasitism (Fig. 7a) [61, 62].

The mechanisms of silver nanoparticles against fungi are not completely elucidated, however according to some studies silver nanoparticles cause changes in the plasma membrane dynamics of fungal cells leading to loss of integrity, increased permeabilization and depolarization. In addition, silver nanoparticles release Ag^+^ ions that interact with oxygen causing an increase in the intracellular levels of reactive oxygen species and an accumulation of hydroxyl radicals. These highly reactive compounds can trigger mitochondrial dysfunction and disruption of ATP synthesis, DNA fragmentation and apoptosis (Fig. 7b) [63, 64].

From the result of our study, we raised the hypothesis of a synergistic effect between the nanoparticles and the capping against *S. sclerotiorum*. The capping of the nanoparticles presented active hydrolytic enzymes of *Trichoderma harzianum* which act by degrading carbohydrates in the cell wall of the phytopathogenic fungi [27, 53, 54]. After fungal cell wall degradation, the silver nanoparticles come into action.

These combined events of capping and silver nanoparticles probably make the fast action of biogenic nanoparticles due to the presence and action of enzymes present in the capping. However, although the effect of the capped nanoparticles on *Sclerotinia sclerotiorum* is a strong indication of this synergy, further studies are needed. Figure 7c shows a hypothetical representation of the synergy between the silver nanoparticles and their capping against *S. sclerotiorum*.

Although several previous studies have reported the potential of biogenic silver nanoparticles for the control of phytopathogens in agriculture, the approach used in the present study enabled evaluation of the importance of the capping for the quality and biological activity of the nanoparticles, opening perspectives for further detailed investigations to explore this feature of biogenic nanoparticles.

#### **Cytotoxic and genotoxic effects of the nanoparticles in cell lines and*Allium cepa***

##### Cytotoxicity evaluation

**Fig. 8:**
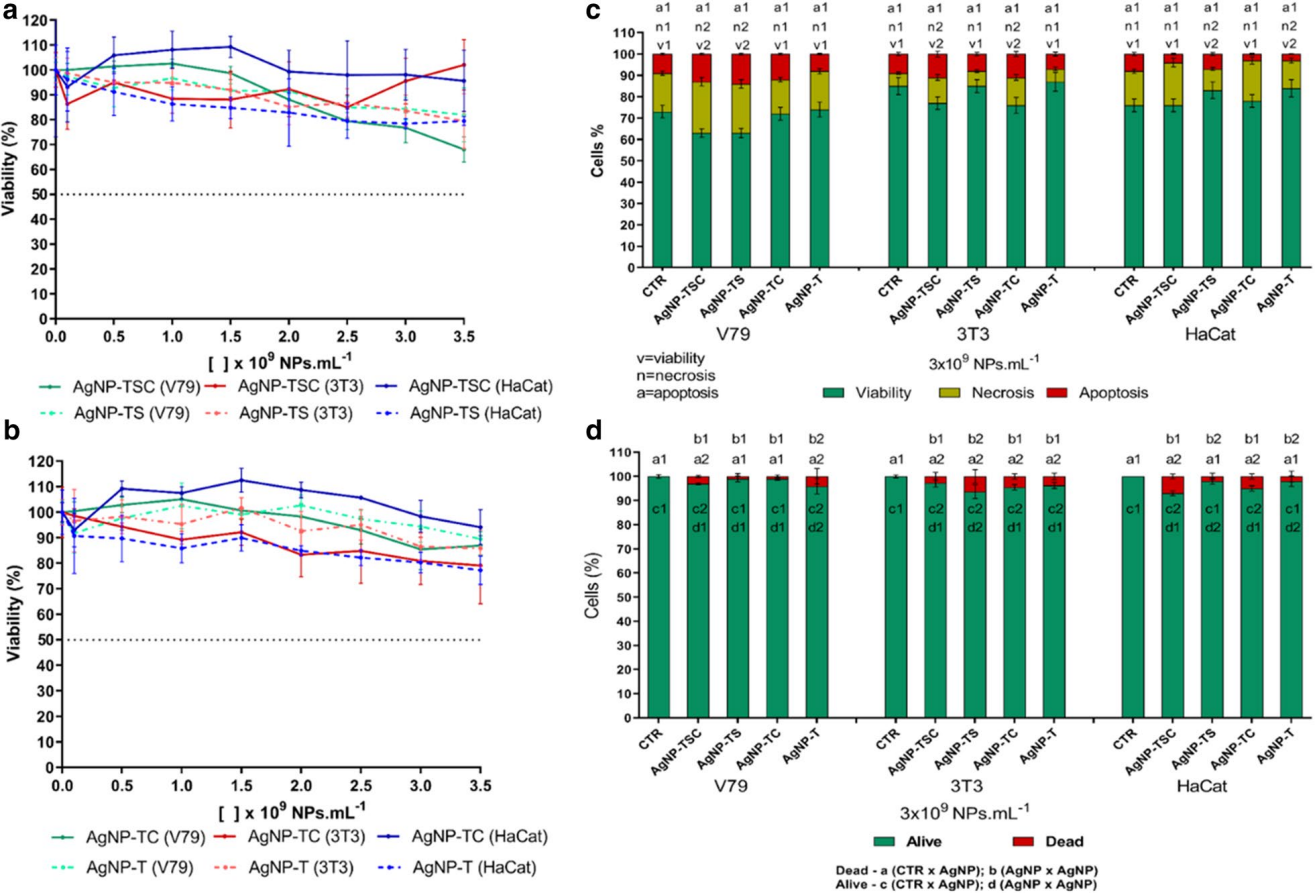
Cytotoxicity evaluation of the AgNP-TSC, AgNP-TS, AgNP-TC, and AgNP-T nanoparticles. **a** MTT assay comparing AgNP-TSC and AgNP-TS; **b** MTT assay comparing AgNP-TC and AgNP-T; **c** imaging cytometry analysis of cell viability, necrosis, and apoptosis; **d** trypan blue exclusion assay. Statistical analysis: different numbers indicate statistically significant difference (p < 0.05)

Use of the MTT assay revealed differences in the cytotoxicity of the capped and uncapped nanoparticles, notably in the HaCat cell line, where both types of uncapped nanoparticles showed greater cytotoxicity. However, IC_50_ values were not reached for any of the samples, indicating low cytotoxicity at the concentrations used in the exposures (Fig. 8a and b). The results of the viability assays using imaging cytometry and the trypan blue test agreed with the MTT assay, with low cell death indices (Fig. 8c and d).

The cytotoxicity of silver nanoparticles can vary greatly, depending on the type of particle and the synthesis process, since it is related to factors such as exposure time, concentration, temperature, particle size, capping, and cell line [15, 39]. An important point is that biogenic silver nanoparticles are usually less cytotoxic than commercial uncapped nanoparticles and silver ions [10]. Skladanowski et al. synthesized silver nanoparticles using *Streptomyces* sp. NH28 and used the MTT assay to evaluate their cytotoxicity towards the L929 mouse fibroblast cell line. No cytotoxicity was observed at the lowest exposure concentrations, at which cell viability was equivalent to that of the control, with IC_50_ only being reached using high concentrations [65]. Although cytotoxicity may be low or absent, it is also important to evaluate the genotoxic effects of new nanoparticles.

##### Genotoxicity evaluation

**Fig. 9:**
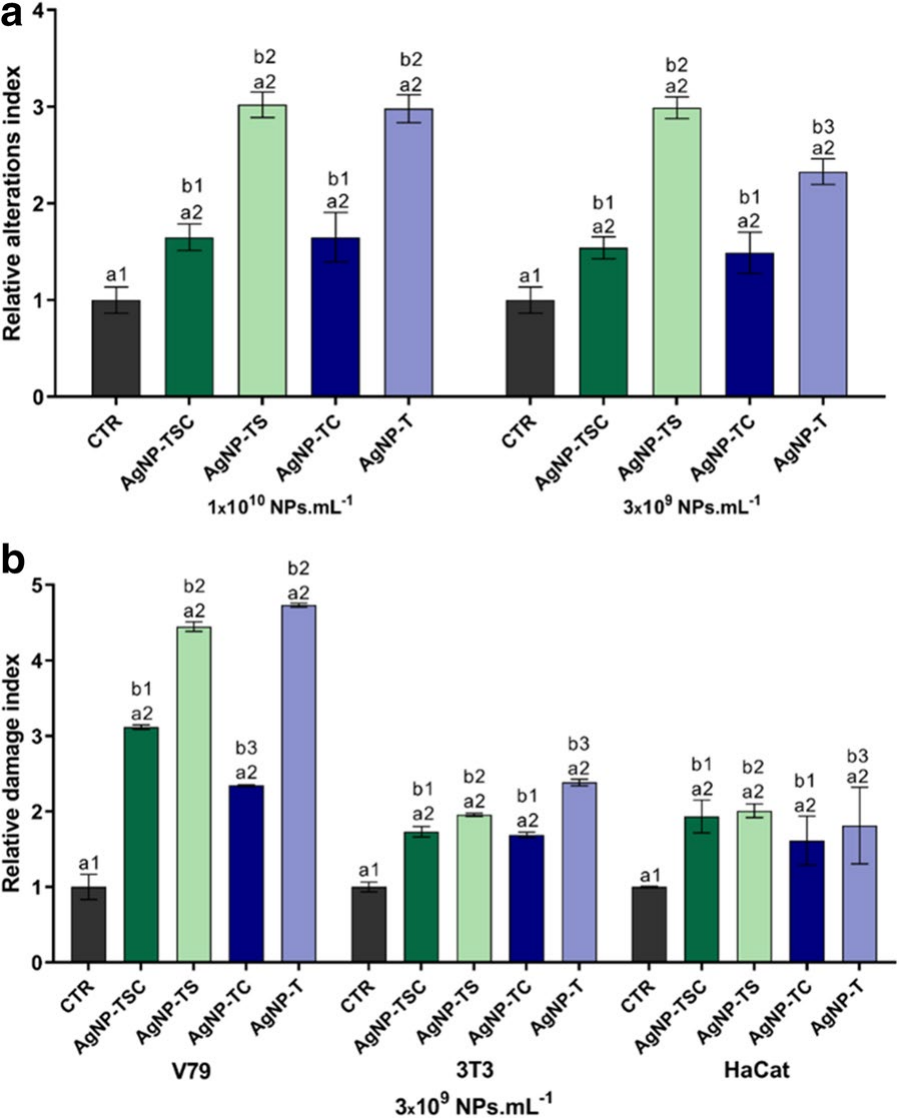
Genotoxicity evaluation of the AgNP-TSC, AgNP-TS, AgNP-TC, and AgNP-T samples. **a** Chromosomal alteration indices obtained using the *Allium cepa* test. **b** DNA damage indices obtained using comet assays. Statistical analysis: different numbers indicate statistically significant difference (p < 0.05)

In the *Allium cepa* genotoxicity assays, the uncapped nanoparticles caused higher alteration indices, compared to the capped nanoparticles, at both exposure concentrations (Fig. 9a). Similar results were obtained in the comet assays, with the uncapped nanoparticles resulting in higher damage indices, compared to the capped nanoparticles (Fig. 9b). The V79 cell line presented higher sensitivity to all the samples, compared to the other cell lines.

The greater genotoxic effects caused by the uncapped nanoparticles could be attributed to the fact that the capping not only increased the stability of the particles, preventing them from aggregating and losing their properties, but also retarded the release of Ag^+^ ions, which are more toxic than nanoparticles composed of Ag^0^ [66]. At non-cytotoxic concentrations, some silver nanoparticles can cause DNA damage, chromosomal aberrations, and possible mutagenic effects [15]. Daphedar and Taranath observed chromosomal aberrations in meristematic root tissues of *Drimia polyantha* exposed to biogenic silver nanoparticles, which were dependent on the nanoparticle concentration and the duration of exposure. It was suggested that internalization of the nanoparticles in the roots could lead to a variety of chromosomal alterations including bridges, multipolar anaphase, delayed division, fusion failures, and C-metaphase, among others [45].

Toxicity studies of biogenic silver nanoparticles have shown varied results, due to the differences in types of biological reducing agents and stabilizers, capping compositions, and synthesis conditions, resulting in the formation of nanoparticles with different characteristics and levels of toxicity [39]. Furthermore, comparisons are hindered by the use of different nanoparticle concentrations and organisms in the exposures. Nonetheless, it is important to evaluate the possibility of toxic effects, in order to ensure responsible and environmentally safe synthesis and application of these nanomaterials. The cytotoxic and genotoxic effects caused by silver nanoparticles can occur following their internalization in cells, causing oxidative stress, membrane damage, alterations of the cell cycle, inflammatory responses, DNA damage, and chromosomal aberrations [58], as well as changes in cell morphology, decreased viability, and cell death by apoptosis and necrosis [67].

### Toxicity of the nanoparticles towards nontarget microorganisms

#### Minimum inhibitory concentration (MIC) for microorganisms of agricultural importance

The MIC values varied according to the type of nanoparticle and the microorganism. Considering the capped nanoparticles, the AgNP-TSC sample presented MIC values of 3.0 × 10^9^, 2.5 × 10^9^, 3.5 × 10^9^, and 1.5 × 10^9^ NPs mL^− 1^ for the microorganisms *Bradyrhizobium japonicum*, *Pseudomonas aeruginosa*, *Bacillus thuringiensis*, and *Beauveria bassiana*, respectively, while the AgNP-TC nanoparticles presented MIC of 3.5 × 10^9^ NPs mL^− 1^ for *Bradyrhizobium japonicum* and *Pseudomonas aeruginosa*, no concentration for *Bacillus thuringiensis*, and 3.0 × 10^9^ NPs mL^− 1^ for *Beauveria bassiana*. In the case of the uncapped nanoparticles, no mortality of the organisms was observed at the exposure concentrations evaluated. A possible reason for the lower toxicity of the uncapped nanoparticles towards the microorganisms was their larger hydrodynamic diameter and consequently lower toxic effects, given that a smaller diameter of silver nanoparticles increases their toxicity to microorganisms [68, 69]. In addition, it is important to consider that these nanoparticles were synthesized by biogenic pathways and present different characteristics in relation to the nanoparticles obtained by conventional synthesis. Removing the capping of biogenic nanoparticles can alter its properties since the capping is responsible for colloidal stability [70].

#### Molecular qPCR analyses of the effects of the nanoparticles on soil microbiota

**Fig. 10:**
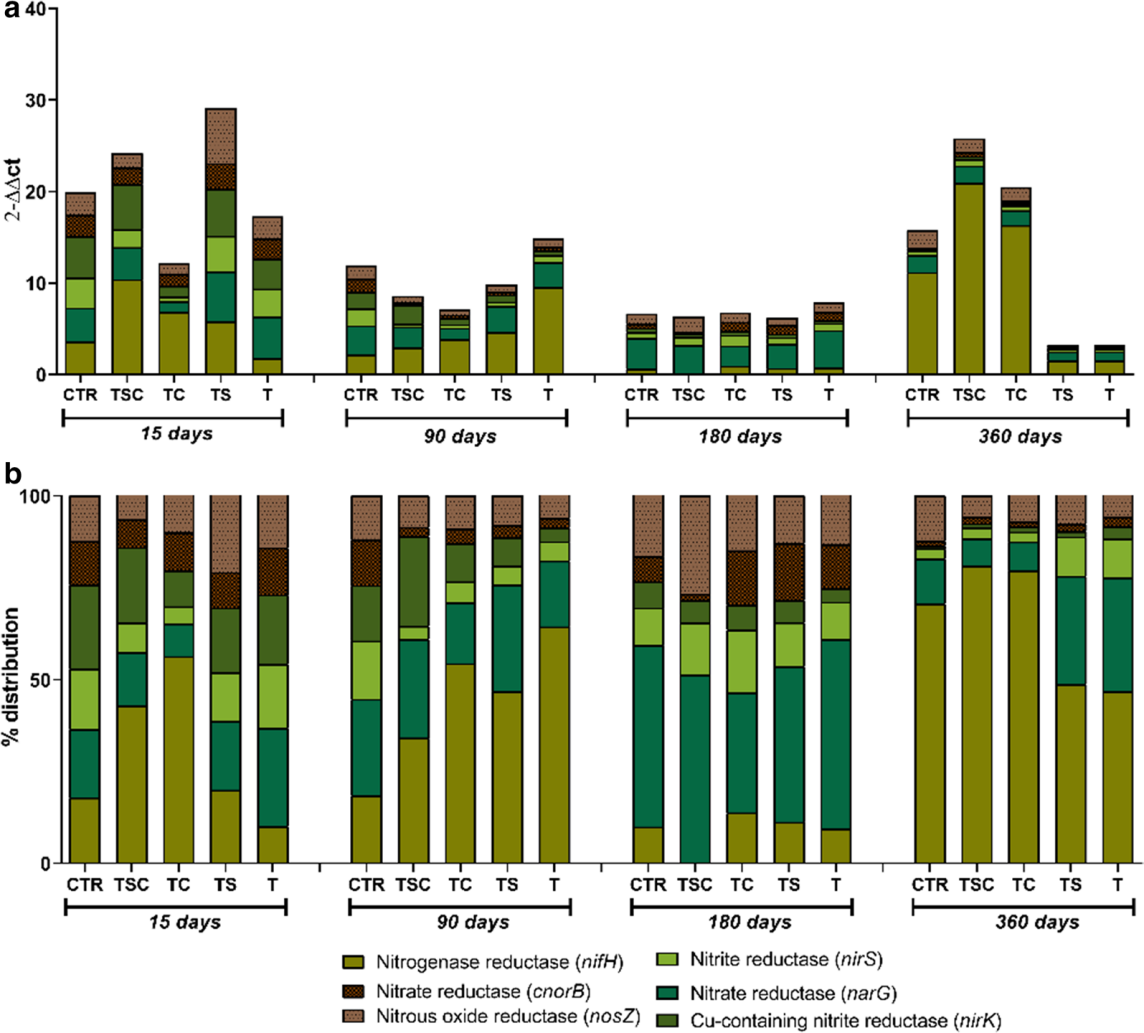
Quantitative molecular analysis of soils exposed to the AgNP-TSC, AgNP-TC, AgNP-TS, and AgNP-T nanoparticles after 15, 90, 180, and 360 days. **a** Quantification calculated using 2^− ΔΔct^. **b** Results for the proportion of each gene analyzed

The soil samples exposed to the capped nanoparticles AgNP-TSC and AgNP-TC showed results closer to the control, in terms of both the quantity and proportions of genes. This could be seen most clearly in the data obtained after 360 days, when lower gene quantification values were obtained for the soils exposed to the uncapped AgNP-TS and AgNP-T nanoparticles (Fig. 10).

After 15 days of exposure, the most evident alteration, relative to the control, was an increase in the proportion of nifH in the soil samples exposed to the AgNP-TSC and AgNP-TC nanoparticles, which was indicative of increased nitrogen fixation. After 90 days, increased nitrogen fixation was also observed for AgNP-TS and AgNP-T, while all four samples showed a reduction of the first phase of denitrification, indicated by the nirS gene. At the same time, AgNP-TSC showed an increase of cnorB, which is associated with the second phase of denitrification, while AgNP-T showed a decrease of this gene. After 180 days, changes were observed for AgNP-TSC, with the disappearance of nifH and decrease of cnorB, while no changes were observed for the other nanoparticles, relative to the control. After 360 days, the soil samples exposed to the AgNP-TSC and AgNP-TC nanoparticles showed greater similarity to the control, compared to AgNP-TS and AgNP-T, indicating that over time, the bacteria population exposed to the capped nanoparticles tended to recover. It should be noted that the control soil without nanoparticles underwent progressive alterations during the course of the trial period.

Grün et al. investigated the effects of silver nanoparticles on soil bacteria, either unfunctionalized or functionalized with amine or carboxyl groups, using quantification of the 16s rRNA, nifH, and amoA genes. Smaller alterations in the distribution of genes were observed for the soil sample exposed to the nanoparticles functionalized with negatively charged carboxyl groups. This was attributed to the effect of the capping, which decreased both the release of ions and direct contact between the silver and the microorganisms, in addition to allowing the formation of bonds with soil cations, which further increased the barrier effect [71]. In other study, VandeVoort and Arai found that nanoparticles capped with polyvinylpyrrolidone (PVP) presented greater affinity with the soil and lower toxicity to denitrification bacteria, compared to uncapped nanoparticles [72].

In the present study, the smaller changes observed for the soils exposed to the capped nanoparticles, especially in terms of the quantities of bacteria, could also be attributed to the presence of the capping. These results agreed with the lower cytotoxic and genotoxic effects of the capped nanoparticles in the assays using in vitro exposure of cell cultures and *Allium cepa*, which could have been associated with possible inhibition of ions release.

## Conclusions

The present study showed that capped biogenic nanoparticles synthesized from different filtrates of the fungus *Trichoderma harzianum* have biological activity in the control of the phytopathogen *Sclerotinia sclerotiorum *in vitro with the effects being attributed to the presence of the capping. Uncapped nanoparticles presented larger hydrodynamic diameters and were ineffective in inhibiting the development of *S. sclerotiorum*. Analysis of the cappings showed that the nanoparticles were capped with biomolecules derived from the filtrate and that they presented specific activities related to the hydrolytic enzymes of *T. harzianum*. The capped nanoparticles were generally less toxic, compared to the uncapped nanoparticles. These findings open perspectives for future studies investigating the composition and importance of the cappings of biogenic nanoparticles employed for the control of phytopathogens in agriculture.

## Data Availability

All data generated or analysed during this study are included in this published article.

## Abbreviations

AgNPs: Silver nanoparticles
AgNP-TSC: Capped silver nanoparticles, with stimulation
AgNP-TC: Capped silver nanoparticles, without stimulation
AgNP-TS: Uncapped silver nanoparticles, with stimulation
AgNP-T: Uncapped silver nanoparticles, without stimulation
MTT: Thiazolyl Blue Tetrazolium Bromide
SDS: Sodium Dodecyl Sulfate
DLS: Dynamic Light Scattering
NTA: Nanoparticle Tracking Analysis
AFM: Atomic Force Microscopy
SDS-PAGE: Sodium Dodecyl Sulfate Polyacrylamide Gel Electrophoresis
NAGase: N-acetylglucosaminidase
MI: Mitotic Index
AI: Alteration Index
MIC: Minimum Inhibitory Concentration
qPCR: Real-Time Polymerase Chain Reaction
ANOVA: One-Way Analysis of Variance
FTIR: Fourier Transform Infrared Spectroscopy
PVP: Polyvinylpyrrolidone
